# The genome sequence of the black-headed gull, *Chroicocephalus ridibundus *(Linnaeus, 1766)

**DOI:** 10.12688/wellcomeopenres.22741.1

**Published:** 2024-07-22

**Authors:** Rosa Lopez Colom, Michelle O’Brien, Natural History Museum Genome Acquisition Lab, Darwin Tree of Life Barcoding collective, Wellcome Sanger Institute Tree of Life Management, Samples and Laboratory team, Wellcome Sanger Institute Scientific Operations: Sequencing Operations, Wellcome Sanger Institute Tree of Life Core Informatics team, Tree of Life Core Informatics collective, Darwin Tree of Life Consortium

**Affiliations:** 1 Wildfowl and Wetlands Trust, Slimbridge, England, UK

**Keywords:** Chroicocephalus ridibundus, black-headed gull, genome sequence, chromosomal, Charadriiformes

## Abstract

We present a genome assembly from an individual male *Chroicocephalus ridibundus* (the black-headed gull; Chordata; Aves; Charadriiformes; Laridae). The genome sequence spans 1,417.60 megabases. Most of the assembly is scaffolded into 33 chromosomal pseudomolecules, including the Z sex chromosome. The mitochondrial genome has also been assembled and is 16.82 kilobases in length.

## Species taxonomy

Eukaryota; Opisthokonta; Metazoa; Eumetazoa; Bilateria; Deuterostomia; Chordata; Craniata; Vertebrata; Gnathostomata; Teleostomi; Euteleostomi; Sarcopterygii; Dipnotetrapodomorpha; Tetrapoda; Amniota; Sauropsida; Sauria; Archelosauria; Archosauria; Dinosauria; Saurischia; Theropoda; Coelurosauria; Aves; Neognathae; Charadriiformes; Laridae; *Chroicocephalus, Chroicocephalus ridibundus* (Linnaeus, 1766) (NCBI:txid1192867).

## Background

The black-headed gull (BHG), *Chroicocephalus ridibundus*, is one of the smallest common gulls within the Laridae family in the UK, reaching a length of 34–37 cm, a wingspan of 100–110 cm, and an average weight of 225–350 g. These gulls typically have a lifespan ranging from 10 to 15 years ( Hume, 2014; Taylor, 2016). BHGs can be observed worldwide with varying plumage that changes depending on the time of year. From March until mid-summer, they exhibit a characteristic dark brown mask or hood, which covers less area compared to a similar but slightly larger Laridae species, the Mediterranean gull ( *Ichthyaetus melanocephalus*) ( Jiguet *et al.*, 2022). They also have a pale-grey back with white ventral colouring, typical of gull species, along with black-edged wings and dark inner wings, and red legs. During the winter months or outside the breeding season, they adopt an overall pale plumage with only a small dark patch on either side of the head ( Hume, 2014).

The BHG is a Holarctic species that easily adapts to different habitats and locations; they can be found in grasslands, wetlands, coastal borders, and marine environments. Although not typically known for being large migratory birds, BHG colonies cover important routes across different flyways; some winter within the breeding range of western Europe and as far south as North Africa, while others winter within the breeding range of south-west Siberia and south-west Asia ( Atkinson *et al.*, 2006). Their medium-distance migratory nature, coupled with the possibility of serving as hosts or reservoir populations, gives them the potential to be effective disseminators of diseases not only within their own taxonomic groups but also across different taxa and geographical regions ( Reed *et al.*, 2003).

In recent years, their colonial breeding behaviour led to extensive mass mortalities of adults and chicks during breeding season in Europe due to the emergence of novel European strains of Highly Pathogenic Avian Influenza (HPAIV) ( Adlhoch *et al.*, 2023). Due to their characteristic breeding philopatry or nest site tenacity ( Piro & Schmitz Ornés, 2021), the recovery ability of seabirds and percentage of return after mass mortality events is an important topic of study to understand the impacts of HPAIV in avian species globally. The completion of the genome sequence of species such as the BHG may contribute to a better understanding of disease dynamics, host factors, and aid in identifying vulnerable populations.

This species is currently classified as of “least concern” within the conservation status categorised by Species of European Conservation and the IUCN Red List; however, within the UK Birds of Conservation, it falls under the “amber” list due to historical declines in population and range trends ( British Trust for Ornithology, 2021).

## Genome sequence report

**Figure 1.**
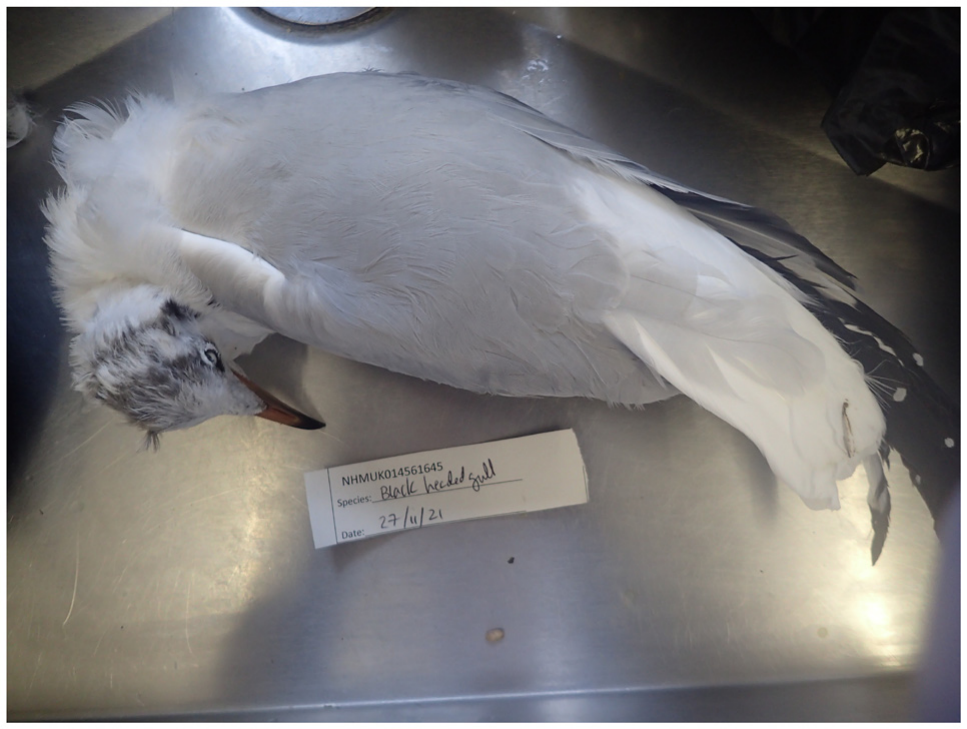
Photograph of the *Chroicocephalus ridibundus* (bChrRid1) specimen used for genome sequencing.

The genome of an adult male *Chroicocephalus ridibundus* ( Figure 1) was sequenced using Pacific Biosciences single-molecule HiFi long reads, generating a total of 28.54 Gb (gigabases) from 2.73 million reads, providing approximately 37-fold coverage. Primary assembly contigs were scaffolded with chromosome conformation Hi-C data, which produced 194.26 Gbp from 1,286.50 million reads, yielding an approximate coverage of 137-fold. Specimen and sequencing information is summarised in Table 1.

**Table 1.** Specimen and sequencing data for *Chroicocephalus ridibundus*.

Project information			
**Study title**	Chroicocephalus ridibundus (black-headed gull)		
**Umbrella BioProject**	PRJEB68288		
**Species**	*Chroicocephalus ridibundus*		
**BioSample**	SAMEA112468038		
**NCBI taxonomy ID**	1192867		
Specimen information			
**Technology**	**ToLID**	**BioSample accession**	**Organism part**
**PacBio long read sequencing**	bChrRid1	SAMEA112468090	muscle
**Hi-C sequencing**	bChrRid1	SAMEA112468090	muscle
**RNA sequencing**	bChrRid1	SAMEA112468090	muscle
Sequencing information			
**Platform**	**Run accession**	**Read count**	**Base count (Gb)**
**Hi-C Illumina NovaSeq 6000**	ERR12259842	1.29e+09	194.26
**PacBio Sequel IIe**	ERR12257418	2.35e+06	22.73
**PacBio Sequel IIe**	ERR12257419	2.73e+06	28.54
**RNA Illumina NovaSeq 6000**	ERR12321242	5.76e+07	8.7

**Figure 2.**
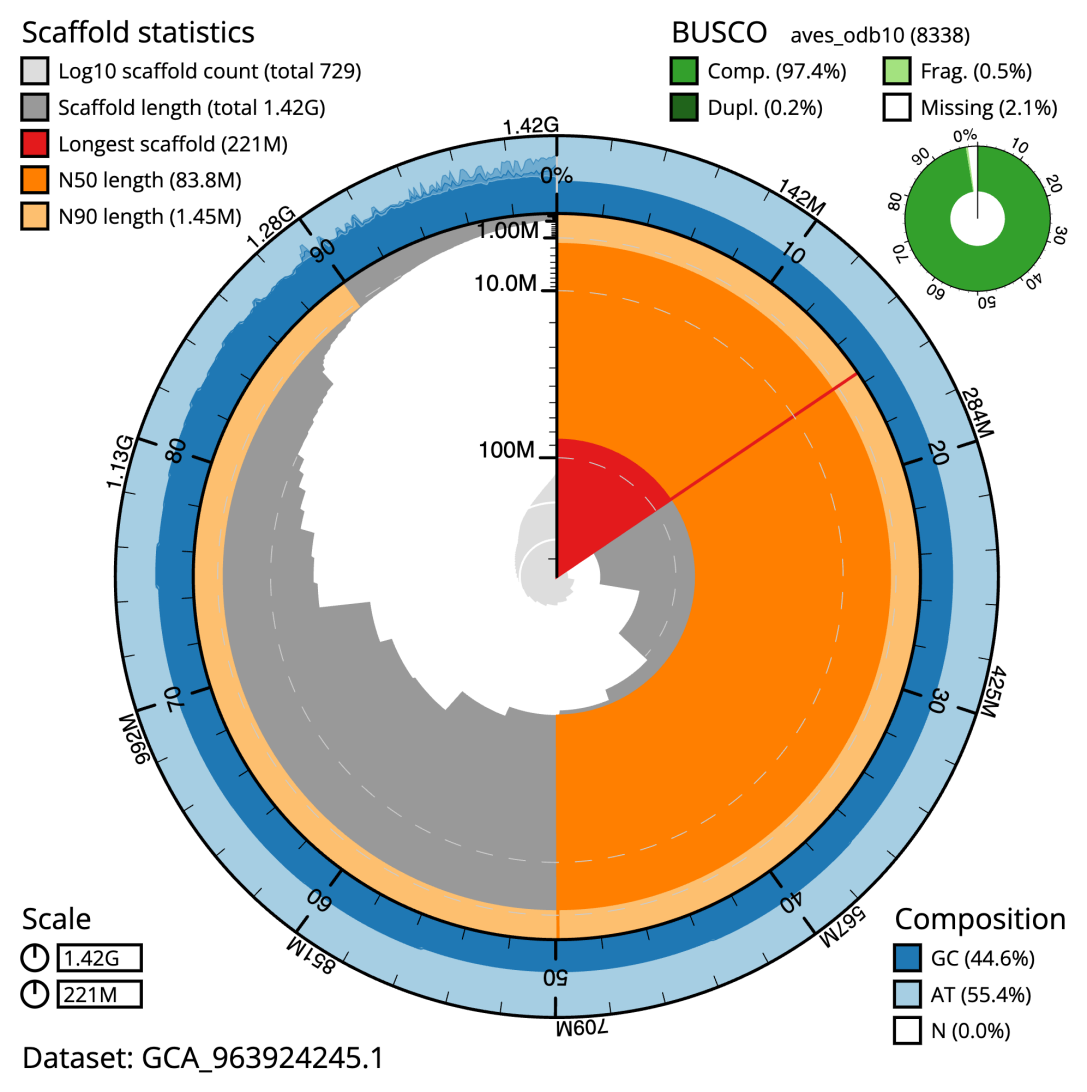
Genome assembly of *Chroicocephalus ridibundus*, bChrRid1.1: metrics. The BlobToolKit snail plot shows N50 metrics and BUSCO gene completeness. The main plot is divided into 1,000 size-ordered bins around the circumference with each bin representing 0.1% of the 1,417,571,123 bp assembly. The distribution of scaffold lengths is shown in dark grey with the plot radius scaled to the longest scaffold present in the assembly (220,911,672 bp, shown in red). Orange and pale-orange arcs show the N50 and N90 scaffold lengths (83,755,488 and 1,452,534 bp), respectively. The pale grey spiral shows the cumulative scaffold count on a log scale with white scale lines showing successive orders of magnitude. The blue and pale-blue area around the outside of the plot shows the distribution of GC, AT and N percentages in the same bins as the inner plot. A summary of complete, fragmented, duplicated and missing BUSCO genes in the aves_odb10 set is shown in the top right. An interactive version of this figure is available at https://blobtoolkit.genomehubs.org/view/Chroicocephalus_ridibundus/dataset/GCA_963924245.1/snail.

**Figure 3.**
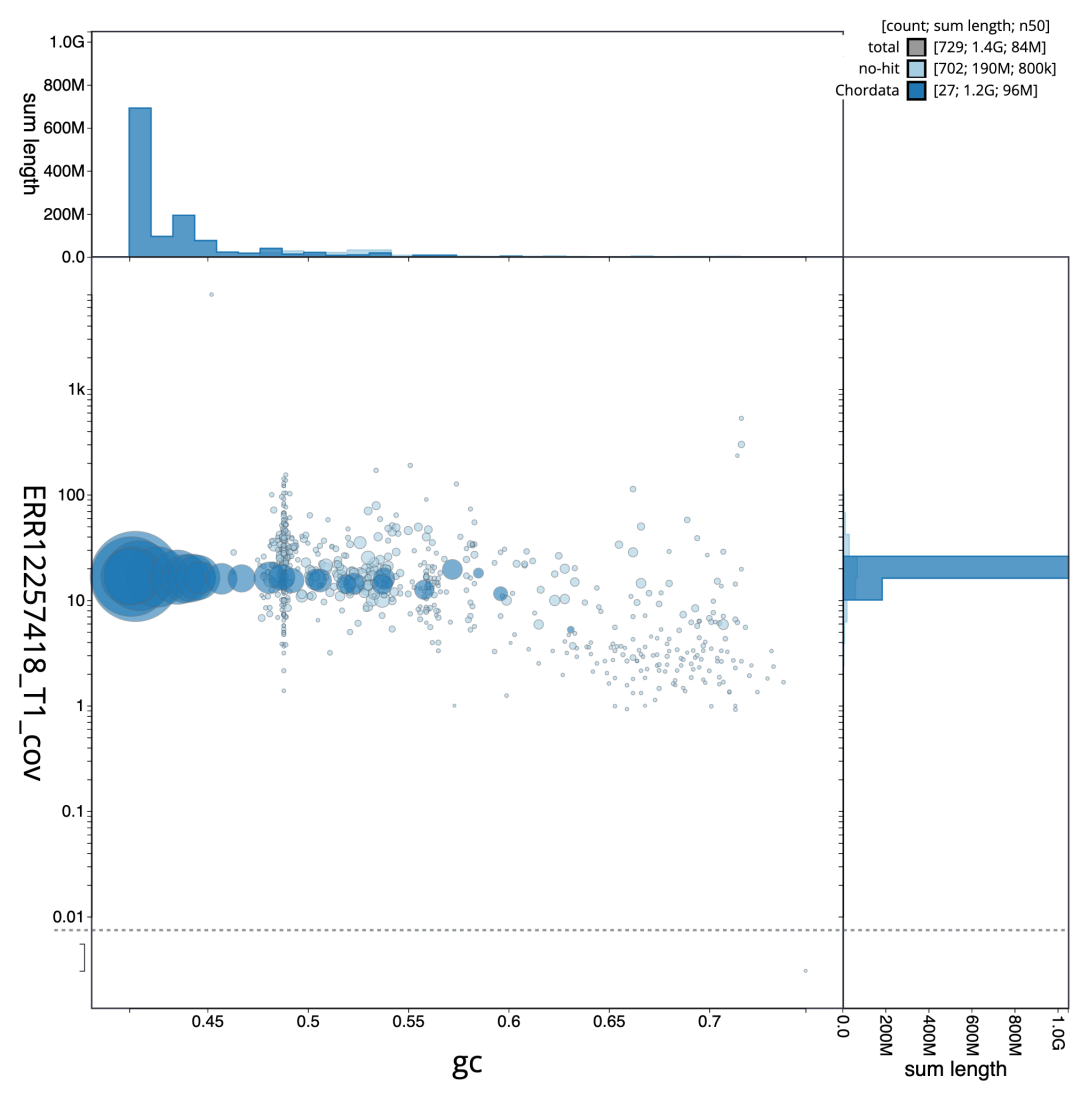
Genome assembly of *Chroicocephalus ridibundus*, bChrRid1.1: BlobToolKit GC-coverage plot. Sequences are coloured by phylum. Circles are sized in proportion to sequence length. Histograms show the distribution of sequence length sum along each axis. An interactive version of this figure is available at https://blobtoolkit.genomehubs.org/view/Chroicocephalus_ridibundus/dataset/GCA_963924245.1/blob.

**Figure 4.**
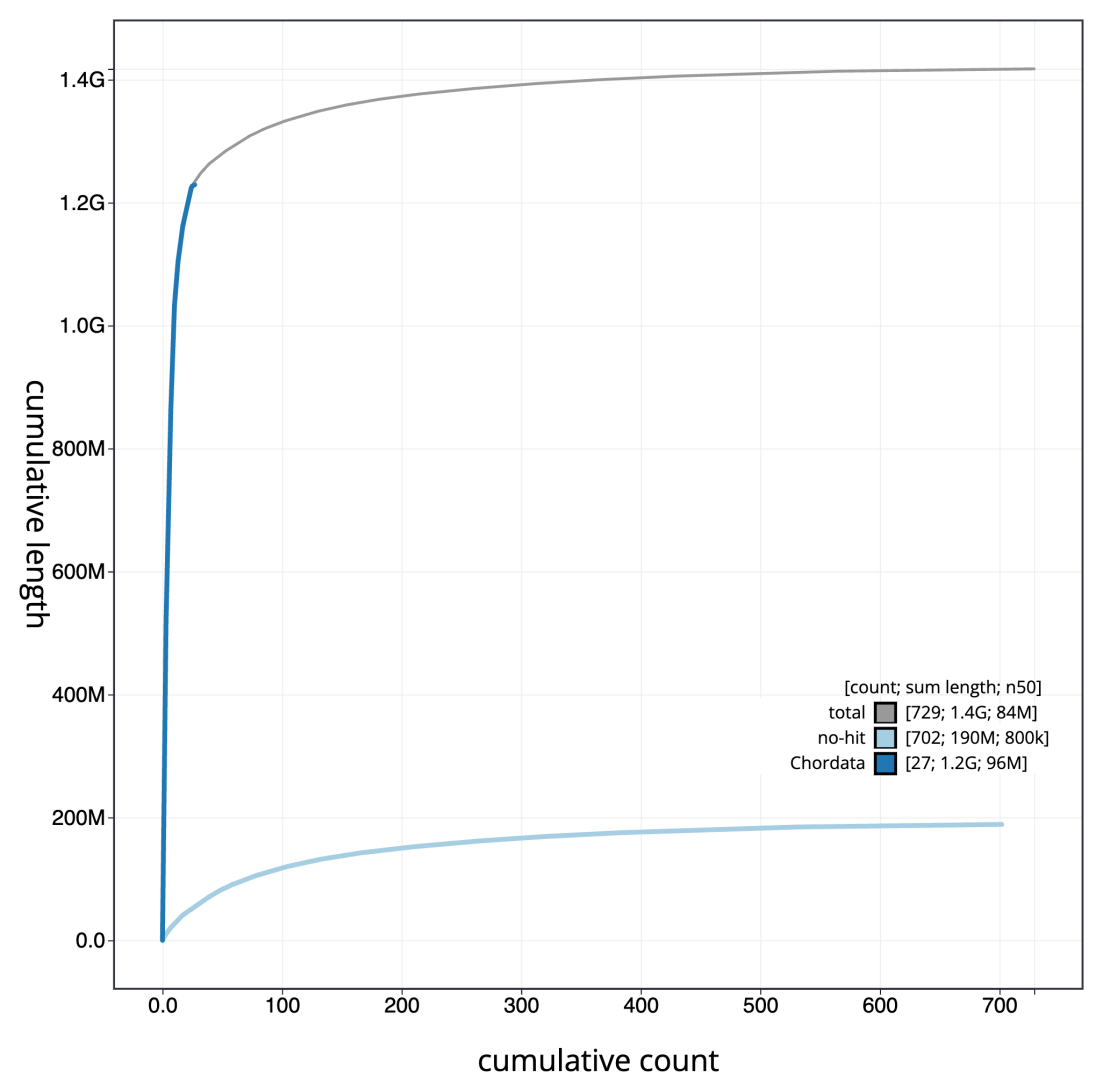
Genome assembly of *Chroicocephalus ridibundus* bChrRid1.1: BlobToolKit cumulative sequence plot. The grey line shows cumulative length for all sequences. Coloured lines show cumulative lengths of sequences assigned to each phylum using the buscogenes taxrule. An interactive version of this figure is available at https://blobtoolkit.genomehubs.org/view/Chroicocephalus_ridibundus/dataset/GCA_963924245.1/cumulative.

**Figure 5.**
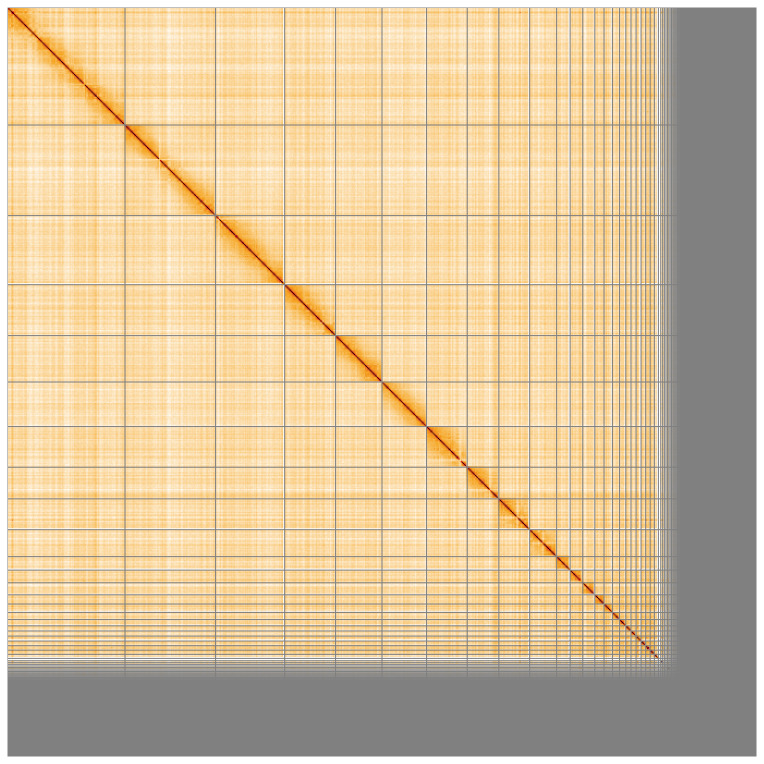
Genome assembly of *Chroicocephalus ridibundus* bChrRid1.1: Hi-C contact map of the bChrRid1.1 assembly, visualised using HiGlass. Chromosomes are shown in order of size from left to right and top to bottom. An interactive version of this figure may be viewed at https://genome-note-higlass.tol.sanger.ac.uk/l/?d=Ed311plvSRuQE2KpKal5Bg.

Manual assembly curation corrected 43 missing joins or mis-joins and one haplotypic duplication, reducing the scaffold number by 3.06%, and increasing the scaffold N50 by 3.26%. The final assembly has a total length of 1,417.60 Mb in 728 sequence scaffolds with a scaffold N50 of 83.8 Mb ( Table 2). The total count of gaps in the scaffolds is 462. The snail plot in Figure 2 provides a summary of the assembly statistics, while the distribution of assembly scaffolds on GC proportion and coverage is shown in Figure 3. The cumulative assembly plot in Figure 4 shows curves for subsets of scaffolds assigned to different phyla. Most (87.31%) of the assembly sequence was assigned to 33 chromosomal-level scaffolds, representing 32 autosomes and the Z sex chromosome. Chromosome-scale scaffolds confirmed by the Hi-C data are named in order of size ( Figure 5; Table 3). The Z chromosome was identified based on alignment with *Gallus gallus* (GCF_016699485.2). While not fully phased, the assembly deposited is of one haplotype. Contigs corresponding to the second haplotype have also been deposited. The mitochondrial genome was also assembled and can be found as a contig within the multifasta file of the genome submission.

**Table 2.** Genome assembly data for *Chroicocephalus ridibundus*, bChrRid1.1.

Genome assembly		
Assembly name	bChrRid1.1	
Assembly accession	GCA_963924245.1	
*Accession of alternate haplotype*	*GCA_963924255.1*	
Span (Mb)	1,417.60	
Number of contigs	1,191	
Contig N50 length (Mb)	4.0	
Number of scaffolds	728	
Scaffold N50 length (Mb)	83.8	
Longest scaffold (Mb)	220.91	
Assembly metrics *		*Benchmark*
Consensus quality (QV)	61.7	*≥ 50*
*k*-mer completeness	100.0%	*≥ 95%*
BUSCO **	C:97.6%[S:97.3%,D:0.3%],F:0.5%,M:1.9%,n:8,338	*C ≥ 95%*
Percentage of assembly mapped to chromosomes	87.31%	*≥ 95%*
Sex chromosomes	Z	*localised homologous pairs*
Organelles	Mitochondrial genome: 16.82 kb	*complete single alleles*

* Assembly metric benchmarks are adapted from column VGP-2020 of “Table 1: Proposed standards and metrics for defining genome assembly quality” from Rhie *et al.* (2021). ** BUSCO scores based on the vertebrata_odb10 BUSCO set using version 5.4.3. C = complete [S = single copy, D = duplicated], F = fragmented, M = missing, n = number of orthologues in comparison. A full set of BUSCO scores is available at https://blobtoolkit.genomehubs.org/view/Chroicocephalus_ridibundus/dataset/GCA_963924245.1/busco.

**Table 3.** Chromosomal pseudomolecules in the genome assembly of *Chroicocephalus ridibundus*, bChrRid1.

INSDC accession	Name	Length (Mb)	GC%
OZ001733.1	1	220.91	41.5
OZ001734.1	2	170.22	41.0
OZ001735.1	3	129.83	41.5
OZ001736.1	4	95.72	42.5
OZ001738.1	5	83.76	41.0
OZ001739.1	6	76.44	43.5
OZ001740.1	7	59.41	44.0
OZ001741.1	8	57.94	44.0
OZ001742.1	9	50.66	44.5
OZ001743.1	10	25.39	44.5
OZ001744.1	11	22.6	45.5
OZ001745.1	12	23.79	48.0
OZ001746.1	13	17.36	46.5
OZ001747.1	14	15.78	48.5
OZ001748.1	15	13.38	49.0
OZ001749.1	16	11.76	50.5
OZ001750.1	17	9.7	54.0
OZ001751.1	18	9.61	50.5
OZ001752.1	19	9.31	52.5
OZ001753.1	20	8.72	53.5
OZ001754.1	21	8.4	57.0
OZ001755.1	22	7.94	56.0
OZ001756.1	23	7.61	52.0
OZ001757.1	24	3.6	59.5
OZ001758.1	25	2.49	52.5
OZ001759.1	26	1.5	58.5
OZ001760.1	27	1.45	52.5
OZ001761.1	28	1.39	60.0
OZ001762.1	29	1.25	61.5
OZ001763.1	30	0.47	63.0
OZ001764.1	31	0.44	63.0
OZ001765.1	32	0.25	66.0
OZ001737.1	Z	87.24	41.5
OZ001766.1	MT	0.02	45.5

The estimated Quality Value (QV) of the final assembly is 61.7 with *k*-mer completeness of 100.0%, and the assembly has a BUSCO v5.4.3 completeness of 97.6% (single = 97.3%, duplicated = 0.3%), using the vertebrata_odb10 reference set ( *n* = 8,338).

Metadata for specimens, BOLD barcode results, spectra estimates, sequencing runs, contaminants and pre-curation assembly statistics are given at https://tolqc.cog.sanger.ac.uk/darwin/birds/Chroicocephalus_ridibundus/.

## Methods

### Sample acquisition

Several small samples of pectoral muscle were collected from a deceased black-headed gull, *Chroicocephalus ridibundus*, specimen ID NHMUK014561645 (ToLID bChrRid1). This Laridae species was collected in Gloucestershire in 2021 as part of a disease surveillance programme carried out by WWT in contribution to the Great Britain Wildlife Health Partnership and kept in cold storage prior to sampling. The specimen was in transition between its summer and winter plumage, losing the characteristic dark face and moulting into a white with grey markings typical of the winter months.

In addition to identification based on morphology, the species taxonomy was verified by DNA barcoding soon after collection, according to the framework developed by Twyford *et al*. (2024). A small sample was dissected from the specimen and stored in ethanol. The tissue was lysed, and the COI marker region was amplified by PCR. Amplicons were sequenced and compared to the BOLD database, confirming the species identification ( Crowley *et al.*, 2023). The standard operating procedures for the Darwin Tree of Life barcoding have been deposited on protocols.io ( Beasley *et al.*, 2023). The remaining parts of the specimen were shipped on dry ice to the Wellcome Sanger Institute (WSI). A DNA barcode was also generated from the PacBio sequencing data at a later stage for sample tracking through the genome production pipeline at the WSI ( Twyford *et al.*, 2024).

### Nucleic acid extraction

The workflow for high molecular weight (HMW) DNA extraction at the Wellcome Sanger Institute (WSI) Tree of Life Core Laboratory includes a sequence of core procedures: sample preparation; sample homogenisation, DNA extraction, fragmentation, and clean-up. In sample preparation, the bChrRid1 sample was weighed and dissected on dry ice ( Jay *et al.*, 2023). For sample homogenisation, muscle tissue was cryogenically disrupted using the Covaris cryoPREP ^®^ Automated Dry Pulverizer ( Narváez-Gómez *et al.*, 2023).

HMW DNA was extracted in the WSI Scientific Operations core using the Automated MagAttract v2 protocol ( Oatley *et al.*, 2023). The DNA was sheared into an average fragment size of 12–20 kb in a Megaruptor 3 system with speed setting 31 ( Bates *et al.*, 2023). Sheared DNA was purified by solid-phase reversible immobilisation ( Strickland *et al.*, 2023): in brief, the method employs a 1.8X ratio of AMPure PB beads to sample to eliminate shorter fragments and concentrate the DNA. The concentration of the sheared and purified DNA was assessed using a Nanodrop spectrophotometer and Qubit Fluorometer using the Qubit dsDNA High Sensitivity Assay kit. Fragment size distribution was evaluated by running the sample on the FemtoPulse system.

RNA was extracted from muscle tissue of bChrRid1 in the Tree of Life Laboratory at the WSI using the RNA Extraction: Automated MagMax™ *mir*Vana protocol ( do Amaral *et al.*, 2023). The RNA concentration was assessed using a Nanodrop spectrophotometer and a Qubit Fluorometer using the Qubit RNA Broad-Range Assay kit. Analysis of the integrity of the RNA was done using the Agilent RNA 6000 Pico Kit and Eukaryotic Total RNA assay.

Protocols developed by the WSI Tree of Life laboratory are publicly available on protocols.io ( Denton *et al.*, 2023).

### Sequencing

Pacific Biosciences HiFi circular consensus DNA sequencing libraries were constructed according to the manufacturers’ instructions. Poly(A) RNA-Seq libraries were constructed using the NEB Ultra II RNA Library Prep kit. DNA and RNA sequencing was performed by the Scientific Operations core at the WSI on Pacific Biosciences Sequel IIe (HiFi) and Illumina NovaSeq 6000 (RNA-Seq) instruments. Hi-C data were also generated from muscle tissue of bChrRid1 using the Arima-HiC v2 kit. The Hi-C sequencing was performed using paired-end sequencing with a read length of 150 bp on the Illumina NovaSeq 6000 instrument.

### Genome assembly, curation and evaluation

***Assembly.*** The original assembly of HiFi reads was performed using Hifiasm ( Cheng *et al.*, 2021) with the --primary option. Haplotypic duplications were identified and removed with purge_dups ( Guan *et al.*, 2020). Hi-C reads are further mapped with bwa-mem2 ( Vasimuddin *et al.*, 2019) to the primary contigs, which are further scaffolded using the provided Hi-C data ( Rao *et al.*, 2014) in YaHS ( Zhou *et al.*, 2023) using the --break option. Scaffolded assemblies are evaluated using Gfastats ( Formenti *et al.*, 2022), BUSCO ( Manni *et al.*, 2021) and MERQURY.FK ( Rhie *et al.*, 2020).

The mitochondrial genome was assembled using MitoHiFi ( Uliano-Silva *et al.*, 2023), which runs MitoFinder ( Allio *et al.*, 2020) and uses these annotations to select the final mitochondrial contig and to ensure the general quality of the sequence.

***Assembly curation.*** The assembly was decontaminated using the Assembly Screen for Cobionts and Contaminants (ASCC) pipeline (article in preparation). Flat files and maps used in curation were generated in TreeVal ( Pointon *et al.*, 2023). Manual curation was primarily conducted using PretextView ( Harry, 2022), with additional insights provided by JBrowse2 ( Diesh *et al.*, 2023) and HiGlass ( Kerpedjiev *et al.*, 2018). Scaffolds were visually inspected and corrected as described by Howe *et al*. (2021). Any identified contamination, missed joins, and mis-joins were corrected, and duplicate sequences were tagged and removed. The sex chromosome was identified by synteny. The entire process is documented at https://gitlab.com/wtsi-grit/rapid-curation (article in preparation).

***Evaluation of the final assembly.*** The final assembly was post-processed and evaluated with the three Nextflow ( Di Tommaso *et al.*, 2017) DSL2 pipelines “sanger-tol/readmapping” ( Surana *et al.*, 2023a), “sanger-tol/genomenote” ( Surana *et al.*, 2023b), and “sanger-tol/blobtoolkit” ( Muffato *et al.*, 2024). The pipeline sanger-tol/readmapping aligns the Hi-C reads with bwa-mem2 ( Vasimuddin *et al.*, 2019) and combines the alignment files with SAMtools ( Danecek *et al.*, 2021). The sanger-tol/genomenote pipeline transforms the Hi-C alignments into a contact map with BEDTools ( Quinlan & Hall, 2010) and the Cooler tool suite ( Abdennur & Mirny, 2020), which is then visualised with HiGlass ( Kerpedjiev *et al.*, 2018). It also provides statistics about the assembly with the NCBI datasets ( Sayers *et al.*, 2024) report, computes *k*-mer completeness and QV consensus quality values with FastK and MERQURY.FK, and a completeness assessment with BUSCO ( Manni *et al.*, 2021).

The sanger-tol/blobtoolkit pipeline is a Nextflow port of the previous Snakemake Blobtoolkit pipeline ( Challis *et al.*, 2020). It aligns the PacBio reads with SAMtools and minimap2 ( Li, 2018) and generates coverage tracks for regions of fixed size. In parallel, it queries the GoaT database ( Challis *et al.*, 2023) to identify all matching BUSCO lineages to run BUSCO ( Manni *et al.*, 2021). For the three domain-level BUSCO lineage, the pipeline aligns the BUSCO genes to the Uniprot Reference Proteomes database ( Bateman *et al.*, 2023) with DIAMOND ( Buchfink *et al.*, 2021) blastp. The genome is also split into chunks according to the density of the BUSCO genes from the closest taxonomically lineage, and each chunk is aligned to the Uniprot Reference Proteomes database with DIAMOND blastx. Genome sequences that have no hit are then chunked with seqtk and aligned to the NT database with blastn ( Altschul *et al.*, 1990). All those outputs are combined with the blobtools suite into a blobdir for visualisation.

The genome assembly and evaluation pipelines were developed using the nf-core tooling ( Ewels *et al.*, 2020), use MultiQC ( Ewels *et al.*, 2016), and make extensive use of the Conda package manager, the Bioconda initiative ( Grüning *et al.*, 2018), the Biocontainers infrastructure ( da Veiga Leprevost *et al.*, 2017), and the Docker ( Merkel, 2014) and Singularity ( Kurtzer *et al.*, 2017) containerisation solutions.

Table 4 contains a list of relevant software tool versions and sources.

**Table 4.** Software tools: versions and sources.

Software tool	Version	Source
BEDTools	2.30.0	https://github.com/arq5x/bedtools2
BLAST	2.14.0	ftp://ftp.ncbi.nlm.nih.gov/blast/executables/ blast+/
BlobToolKit	4.3.7	https://github.com/blobtoolkit/blobtoolkit
BUSCO	5.4.3 and 5.5.0	https://gitlab.com/ezlab/busco
bwa-mem2	2.2.1	https://github.com/bwa-mem2/bwa-mem2
Cooler	0.8.11	https://github.com/open2c/cooler
DIAMOND	2.1.8	https://github.com/bbuchfink/diamond
fasta_windows	0.2.4	https://github.com/tolkit/fasta_windows
FastK	427104ea91c78c3b8b8b49f1a7d6bbeaa869ba1c	https://github.com/thegenemyers/FASTK
Gfastats	1.3.6	https://github.com/vgl-hub/gfastats
GoaT CLI	0.2.5	https://github.com/genomehubs/goat-cli
Hifiasm	0.19.5-r587	https://github.com/chhylp123/hifiasm
HiGlass	44086069ee7d4d3f6f3f0012569789ec138f42b84aa 44357826c0b6753eb28de	https://github.com/higlass/higlass
Merqury.FK	d00d98157618f4e8d1a9190026b19b471055b22e	https://github.com/thegenemyers/MERQURY.FK
MitoHiFi	3	https://github.com/marcelauliano/MitoHiFi
MultiQC	1.14, 1.17, and 1.18	https://github.com/MultiQC/MultiQC
NCBI Datasets	15.12.0	https://github.com/ncbi/datasets
Nextflow	23.04.0-5857	https://github.com/nextflow-io/nextflow
PretextView	0.2	https://github.com/sanger-tol/PretextView
purge_dups	1.2.5	https://github.com/dfguan/purge_dups
samtools	1.16.1, 1.17, and 1.18	https://github.com/samtools/samtools
sanger-tol/ascc	-	https://github.com/sanger-tol/ascc
sanger-tol/ genomenote	1.1.1	https://github.com/sanger-tol/genomenote
sanger-tol/ readmapping	1.2.1	https://github.com/sanger-tol/readmapping
Seqtk	1.3	https://github.com/lh3/seqtk
Singularity	3.9.0	https://github.com/sylabs/singularity
TreeVal	1.0.0	https://github.com/sanger-tol/treeval
YaHS	1.2a.2	https://github.com/c-zhou/yahs

### Wellcome Sanger Institute – Legal and Governance

The materials that have contributed to this genome note have been supplied by a Darwin Tree of Life Partner. The submission of materials by a Darwin Tree of Life Partner is subject to the **‘Darwin Tree of Life Project Sampling Code of Practice’**, which can be found in full on the Darwin Tree of Life website here. By agreeing with and signing up to the Sampling Code of Practice, the Darwin Tree of Life Partner agrees they will meet the legal and ethical requirements and standards set out within this document in respect of all samples acquired for, and supplied to, the Darwin Tree of Life Project.

Further, the Wellcome Sanger Institute employs a process whereby due diligence is carried out proportionate to the nature of the materials themselves, and the circumstances under which they have been/are to be collected and provided for use. The purpose of this is to address and mitigate any potential legal and/or ethical implications of receipt and use of the materials as part of the research project, and to ensure that in doing so we align with best practice wherever possible. The overarching areas of consideration are:

- Ethical review of provenance and sourcing of the material
- Legality of collection, transfer and use (national and international)

Each transfer of samples is further undertaken according to a Research Collaboration Agreement or Material Transfer Agreement entered into by the Darwin Tree of Life Partner, Genome Research Limited (operating as the Wellcome Sanger Institute), and in some circumstances other Darwin Tree of Life collaborators.

## Data Availability

European Nucleotide Archive: Chroicocephalus ridibundus (black-headed gull). Accession number PRJEB68288; https://identifiers.org/ena.embl/PRJEB68288 ( Wellcome Sanger Institute, 2024). The genome sequence is released openly for reuse. The *Chroicocephalus ridibundus* genome sequencing initiative is part of the Darwin Tree of Life (DToL) project. All raw sequence data and the assembly have been deposited in INSDC databases. The genome will be annotated using available RNA-Seq data and presented through the Ensembl pipeline at the European Bioinformatics Institute. Raw data and assembly accession identifiers are reported in Table 1 and Table 2.

## Author information

Members of the Natural History Museum Genome Acquisition Lab are listed here: https://doi.org/10.5281/zenodo.7139035.

Members of the Darwin Tree of Life Barcoding collective are listed here: https://doi.org/10.5281/zenodo.4893703.

Members of the Wellcome Sanger Institute Tree of Life Management, Samples and Laboratory team are listed here: https://doi.org/10.5281/zenodo.10066175.

Members of Wellcome Sanger Institute Scientific Operations: Sequencing Operations are listed here: https://doi.org/10.5281/zenodo.10043364.

Members of the Wellcome Sanger Institute Tree of Life Core Informatics team are listed here: https://doi.org/10.5281/zenodo.10066637.

Members of the Tree of Life Core Informatics collective are listed here: https://doi.org/10.5281/zenodo.5013541.

Members of the Darwin Tree of Life Consortium are listed here: https://doi.org/10.5281/zenodo.4783558.

## References

[ref-1] Abdennur N Mirny LA : Cooler: scalable storage for Hi-C data and other genomically labeled arrays. *Bioinformatics.* 2020;36(1):311–316. 10.1093/bioinformatics/btz540 31290943 PMC8205516

[ref-2] Adlhoch C Fusaro A Gonzales JL : Avian influenza overview April – June 2023. *EFSA J.* 2023;21(7):311–316, e08191. 10.2903/j.efsa.2023.8191 37485254 PMC10358191

[ref-3] Allio R Schomaker-Bastos A Romiguier J : MitoFinder: efficient automated large-scale extraction of mitogenomic data in target enrichment phylogenomics. *Mol Ecol Resour.* 2020;20(4):892–905. 10.1111/1755-0998.13160 32243090 PMC7497042

[ref-4] Altschul SF Gish W Miller W : Basic Local Alignment Search Tool. *J Mol Biol.* 1990;215(3):403–410. 10.1016/S0022-2836(05)80360-2 2231712

[ref-5] Atkinson PW Clark JA Delany S : Urgent preliminary assessment of ornithological data relevant to the spread of avian influenza in Europe. Report to the European commission.2006. Reference Source

[ref-6] Bateman A Martin M-J Orchard S : UniProt: the universal protein knowledgebase in 2023. *Nucleic Acids Res.* 2023;51(D1):D523–D531. 10.1093/nar/gkac1052 36408920 PMC9825514

[ref-7] Bates A Clayton-Lucey I Howard C : Sanger Tree of Life HMW DNA fragmentation: diagenode Megaruptor®3 for LI PacBio. *protocols.io.* 2023. 10.17504/protocols.io.81wgbxzq3lpk/v1

[ref-8] Beasley J Uhl R Forrest LL : DNA barcoding SOPs for the Darwin Tree of Life project. *protocols.io.* 2023; [Accessed 25 June 2024]. 10.17504/protocols.io.261ged91jv47/v1

[ref-9] British Trust for Ornithology: Birds of Conservation Concern. BTO - British Trust for Ornithology,2021; [Accessed 17 June 2024]. Reference Source

[ref-10] Buchfink B Reuter K Drost HG : Sensitive protein alignments at Tree-of-Life scale using DIAMOND. *Nat Methods.* 2021;18(4):366–368. 10.1038/s41592-021-01101-x 33828273 PMC8026399

[ref-11] Challis R Kumar S Sotero-Caio C : Genomes on a Tree (GoaT): a versatile, scalable search engine for genomic and sequencing project metadata across the eukaryotic Tree of Life [version 1; peer review: 2 approved]. *Wellcome Open Res.* 2023;8:24. 10.12688/wellcomeopenres.18658.1 36864925 PMC9971660

[ref-12] Challis R Richards E Rajan J : BlobToolKit – interactive quality assessment of genome assemblies. *G3 (Bethesda).* 2020;10(4):1361–1374. 10.1534/g3.119.400908 32071071 PMC7144090

[ref-13] Cheng H Concepcion GT Feng X : Haplotype-resolved *de novo* assembly using phased assembly graphs with hifiasm. *Nat Methods.* 2021;18(2):170–175. 10.1038/s41592-020-01056-5 33526886 PMC7961889

[ref-14] Crowley L Allen H Barnes I : A sampling strategy for genome sequencing the British terrestrial arthropod fauna [version 1; peer review: 2 approved]. *Wellcome Open Res.* 2023;8:123. 10.12688/wellcomeopenres.18925.1 37408610 PMC10318377

[ref-15] da Veiga Leprevost F Grüning BA Alves Aflitos S : BioContainers: an open-source and community-driven framework for software standardization. *Bioinformatics.* 2017;33(16):2580–2582. 10.1093/bioinformatics/btx192 28379341 PMC5870671

[ref-16] Danecek P Bonfield JK Liddle J : Twelve years of SAMtools and BCFtools. *Gigascience.* 2021;10(2): giab008. 10.1093/gigascience/giab008 33590861 PMC7931819

[ref-17] Denton A Yatsenko H Jay J : Sanger Tree of Life wet laboratory protocol collection V.1. *protocols.io.* 2023. 10.17504/protocols.io.8epv5xxy6g1b/v1

[ref-18] Di Tommaso P Chatzou M Floden EW : Nextflow enables reproducible computational workflows. *Nat Biotechnol.* 2017;35(4):316–319. 10.1038/nbt.3820 28398311

[ref-19] Diesh C Stevens GJ Xie P : JBrowse 2: a modular genome browser with views of synteny and structural variation. *Genome Biol.* 2023;24(1): 74. 10.1186/s13059-023-02914-z 37069644 PMC10108523

[ref-20] do Amaral RJV Bates A Denton A : Sanger Tree of Life RNA extraction: automated MagMax ^TM^ mirVana. *protocols.io.* 2023. 10.17504/protocols.io.6qpvr36n3vmk/v1

[ref-22] Ewels P Magnusson M Lundin S : MultiQC: summarize analysis results for multiple tools and samples in a single report. *Bioinformatics.* 2016;32(19):3047–3048. 10.1093/bioinformatics/btw354 27312411 PMC5039924

[ref-21] Ewels PA Peltzer A Fillinger S : The nf-core framework for community-curated bioinformatics pipelines. *Nat Biotechnol.* 2020;38(3):276–278. 10.1038/s41587-020-0439-x 32055031

[ref-23] Formenti G Abueg L Brajuka A : Gfastats: conversion, evaluation and manipulation of genome sequences using assembly graphs. *Bioinformatics.* 2022;38(17):4214–4216. 10.1093/bioinformatics/btac460 35799367 PMC9438950

[ref-24] Grüning B Dale R Sjödin A : Bioconda: sustainable and comprehensive software distribution for the life sciences. *Nat Methods.* 2018;15(7):475–476. 10.1038/s41592-018-0046-7 29967506 PMC11070151

[ref-25] Guan D McCarthy SA Wood J : Identifying and removing haplotypic duplication in primary genome assemblies. *Bioinformatics.* 2020;36(9):2896–2898. 10.1093/bioinformatics/btaa025 31971576 PMC7203741

[ref-26] Harry E : PretextView (Paired REad TEXTure Viewer): a desktop application for viewing pretext contact maps.2022; [Accessed 19 October 2022]. Reference Source

[ref-27] Howe K Chow W Collins J : Significantly improving the quality of genome assemblies through curation. *Gigascience.* 2021;10(1): giaa153. 10.1093/gigascience/giaa153 33420778 PMC7794651

[ref-28] Hume R : RSPB birds of Britain and Europe. 4th ed. London: DK,2014. Reference Source

[ref-29] Jay J Yatsenko H Narváez-Gómez JP : Sanger Tree of Life sample preparation: triage and dissection. *protocols.io.* 2023. 10.17504/protocols.io.x54v9prmqg3e/v1

[ref-30] Jiguet F Muusse M Dubois PJ : Gulls of Europe, North Africa, and the Middle East.Princeton University Press,2022. Reference Source

[ref-31] Kerpedjiev P Abdennur N Lekschas F : HiGlass: web-based visual exploration and analysis of genome interaction maps. *Genome Biol.* 2018;19(1): 125. 10.1186/s13059-018-1486-1 30143029 PMC6109259

[ref-32] Kurtzer GM Sochat V Bauer MW : Singularity: scientific containers for mobility of compute. *PLoS One.* 2017;12(5): e0177459. 10.1371/journal.pone.0177459 28494014 PMC5426675

[ref-33] Li H : Minimap2: pairwise alignment for nucleotide sequences. *Bioinformatics.* 2018;34(18):3094–3100. 10.1093/bioinformatics/bty191 29750242 PMC6137996

[ref-34] Manni M Berkeley MR Seppey M : BUSCO update: novel and streamlined workflows along with broader and deeper phylogenetic coverage for scoring of eukaryotic, prokaryotic, and viral genomes. *Mol Biol Evol.* 2021;38(10):4647–4654. 10.1093/molbev/msab199 34320186 PMC8476166

[ref-35] Merkel D : Docker: lightweight Linux containers for consistent development and deployment. *Linux Journal.* 2014;2014(239):2. 10.5555/2600239.2600241

[ref-36] Muffato M Butt Z Challis R : sanger-tol/blobtoolkit: v0.3.0 – Poliwag.2024. 10.5281/zenodo.10649272

[ref-37] Narváez-Gómez JP Mbye H Oatley G : Sanger Tree of Life sample homogenisation: covaris CryoPREP® automated dry pulverizer V.1. *protocols.io.* 2023. 10.17504/protocols.io.eq2lyjp5qlx9/v1

[ref-38] Oatley G Denton A Howard C : Sanger Tree of Life HMW DNA extraction: automated MagAttract v.2. *protocols.io.* 2023. 10.17504/protocols.io.kxygx3y4dg8j/v1

[ref-39] Piro S Schmitz Ornés A : Nest site tenacity and mate fidelity in the Black-headed Gull ( *Chroicocephalus ridibundus*). *Avian Res.* 2021;12(1): 63. 10.1186/s40657-021-00300-6

[ref-40] Pointon DL Eagles W Sims Y : sanger-tol/treeval v1.0.0 – Ancient Atlantis (H1).2023. 10.5281/zenodo.10047653

[ref-41] Quinlan AR Hall IM : BEDTools: a flexible suite of utilities for comparing genomic features. *Bioinformatics.* 2010;26(6):841–842. 10.1093/bioinformatics/btq033 20110278 PMC2832824

[ref-42] Rao SSP Huntley MH Durand NC : A 3D map of the human genome at kilobase resolution reveals principles of chromatin looping. *Cell.* 2014;159(7):1665–1680. 10.1016/j.cell.2014.11.021 25497547 PMC5635824

[ref-43] Reed KD Meece JK Henkel JS : Birds, migration and emerging zoonoses: West Nile Virus, lyme disease, influenza A and enteropathogens. *Clin Med Res.* 2003;1(1):5–12. 10.3121/cmr.1.1.5 15931279 PMC1069015

[ref-44] Rhie A McCarthy SA Fedrigo O : Towards complete and error-free genome assemblies of all vertebrate species. *Nature.* 2021;592(7856):737–746. 10.1038/s41586-021-03451-0 33911273 PMC8081667

[ref-45] Rhie A Walenz BP Koren S : Merqury: reference-free quality, completeness, and phasing assessment for genome assemblies. *Genome Biol.* 2020;21(1): 245. 10.1186/s13059-020-02134-9 32928274 PMC7488777

[ref-46] Sayers EW Cavanaugh M Clark K : GenBank 2024 update. *Nucleic Acids Res.* 2024;52(D1):D134–D137. 10.1093/nar/gkad903 37889039 PMC10767886

[ref-47] Strickland M Cornwell C Howard C : Sanger tree of life fragmented DNA clean up: manual SPRI. *protocols.io.* 2023. 10.17504/protocols.io.kxygx3y1dg8j/v1

[ref-48] Surana P Muffato M Qi G : sanger-tol/readmapping: sanger-tol/readmapping v1.1.0 - Hebridean Black (1.1.0). *Zenodo.* 2023a. 10.5281/zenodo.7755669

[ref-49] Surana P Muffato M Sadasivan Baby C : sanger-tol/genomenote (v1.0.dev). *Zenodo.* 2023b. 10.5281/zenodo.6785935

[ref-50] Taylor M : RSPB british birdfinder.Bloomsbury Publishing,2016. Reference Source

[ref-51] Twyford AD Beasley J Barnes I : A DNA barcoding framework for taxonomic verification in the Darwin Tree of Life Project [version 1; peer review: awaiting peer review]. *Wellcome Open Res.* 2024;9:339. 10.12688/wellcomeopenres.21143.1 39386966 PMC11462125

[ref-52] Uliano-Silva M Ferreira JGRN Krasheninnikova K : MitoHiFi: a python pipeline for mitochondrial genome assembly from PacBio high fidelity reads. *BMC Bioinformatics.* 2023;24(1): 288. 10.1186/s12859-023-05385-y 37464285 PMC10354987

[ref-53] Vasimuddin M Misra S Li H : Efficient architecture-aware acceleration of bwa-mem for multicore systems.In: *2019 IEEE International Parallel and Distributed Processing Symposium (IPDPS).*IEEE.2019;314–324. 10.1109/IPDPS.2019.00041

[ref-55] Wellcome Sanger Institute: The genome sequence of the black-headed gull, *chroicocephalus ridibundus* (linnaeus, 1766). European Nucleotide Archive. [dataset]. accession number PRJEB68288.2024. 10.12688/wellcomeopenres.22741.2 PMC11933786 40134894

[ref-54] Zhou C McCarthy SA Durbin R : YaHS: yet another Hi-C scaffolding tool. *Bioinformatics.* 2023;39(1): btac808. 10.1093/bioinformatics/btac808 36525368 PMC9848053

