# Supplementary material for: The genome sequence of the black-headed gull, Chroicocephalus ridibundus (Linnaeus, 1766)
Source: Wellcome Open Res. 2025 Feb 21;9:399. Originally published 2024 Jul 22. [Version 2] doi: 10.12688/wellcomeopenres.22741.2 (PMC11933786; doi:10.12688/wellcomeopenres.22741.2)
Supplement: Peer review history

# Reviewer report: Reviewer response for version 2

**DOI:** 10.21956/wellcomeopenres.26278.r119989

**Published:** 2025-03-24

**Authors:** Yang Liu

**Affiliations:** 1 School of Ecology, Sun Yat-sen University, Shenzhen, China

This manuscript reports a chromosome-level genome assembly for the black-headed gull ( *Chroicocephalus ridibundus*). Although the authors provide comprehensive methodological details, the need to create this genomic resource is not fully clear.

 1. In the background section, the authors mention that completing of the genome sequence of the BHG could enhance our understanding of disease dynamics. While HPAIV has been associated with mass mortalities in BHG populations, there is no direct evidence linking the disease to host genome. Furthermore, the authors did not provide a clear rationale for how assembling the BHG genome would elucidate the dynamics of HPAIV infection.

 2. In the genome annotation report, the described BUSCO results do not align with the complete BUSCO assessment scores at

https://blobtoolkit.genomehubs.org/view/Chroicocephalus_ridibundus/dataset/GCA_963924245.1/busco. Furthermore, the vertebrata_odb10 dataset comprises 3,354 single-copy orthologs, not 8,338.

Are sufficient details of methods and materials provided to allow replication by others?

Yes

Is the rationale for creating the dataset(s) clearly described?

Partly

Are the datasets clearly presented in a useable and accessible format?

Yes

Are the protocols appropriate and is the work technically sound?

Yes

Reviewer Expertise:

Evolutionary biology, avian genomics

I confirm that I have read this submission and believe that I have an appropriate level of expertise to confirm that it is of an acceptable scientific standard.

## References

[1] Dataset. *BlobToolKit* . Reference source

# Reviewer report: Reviewer response for version 1

**DOI:** 10.21956/wellcomeopenres.25044.r97394

**Published:** 2024-09-24

**Authors:** Zhuo-Cheng Hou

**Affiliations:** 1 College of Animal Science and Technology, China Agricultural University Education Foundation, Beijing, Beijing, China

This resources are useful for genomics, tree of life.

**However, there are several minor issues:**

 (1) genome assembly details, example, main commands should be provided for each major assembly software.  This is the necessary step for genome assembly project, which could be reproduced by others. 

 (2) Why do some chromosomes have so much GC contents than others? It is not the single chromosome, but several chromosomes have much higher GC than 45%. Do you check the raw sequencing data? Or give some potential explains.

Are sufficient details of methods and materials provided to allow replication by others?

Partly

Is the rationale for creating the dataset(s) clearly described?

Yes

Are the datasets clearly presented in a useable and accessible format?

Yes

Are the protocols appropriate and is the work technically sound?

Yes

Reviewer Expertise:

genomics and genetics

I confirm that I have read this submission and believe that I have an appropriate level of expertise to confirm that it is of an acceptable scientific standard, however I have significant reservations, as outlined above.

# Response

**Published:** 2025-02-15

**Authors:** Tree of Life Team Sanger

**Affiliations:** Wellcome Trust Sanger Institute, UK

Thank you for reviewing this data notes. Please see the changes that have been made to the second version of the data note. 

 The GC-rich nature of bird microchromsomes is something that we observe frequently. This phenomenon has been described here: https://www.pnas.org/doi/10.1073/pnas.2112494118.

# Reviewer report: Reviewer response for version 1

**DOI:** 10.21956/wellcomeopenres.25044.r94106

**Published:** 2024-09-11

**Authors:** Phred Benham

**Affiliations:** 1 Department of Biology, University of Massachusetts Amherst (Ringgold ID: 117236), Amherst, Massachusetts, USA

The authors report a highly contiguous draft assembly of the Black-headed Gull. The rationale for generating the assembly is sound. The authors combine PacBio and Hi-C methods to assemble contigs into 33 chromosomal pseudo molecules. In general sequencing and assembly methods are sound and follow latest best practices for assembling avian genomes. 

**I have only a few comments that might improve the manuscript:** 

- First, there are at least 3-4 other high quality genomes within Laridae (e.g. Kittiwake, Lesser Black-backed Gull). It would be nice to see how the black-headed gull genome compares to these other genomes. A synteny plot with other gulls would be one nice addition for making this comparison.
- Second, the methods describe extraction and sequencing of RNA, but there is not mention of what this data is used for in the genome sequence report or methods. Presumably this is for annotation? Please describe annotation details in methods/results if available.
- Third, some additional details on library prep would be useful such as conditions of different enzymatic steps. At least state whether steps follow manufacturer's protocols.

**Minor comments:**

- In genome sequence report section. I believe you used the aves_odb10 BUSCO database and not the vertebrata_odb10 database.
- In Fig. 3 can you fix the y-axis to something more meaningful. I have no idea what ERR1225... means.

Are sufficient details of methods and materials provided to allow replication by others?

Yes

Is the rationale for creating the dataset(s) clearly described?

Yes

Are the datasets clearly presented in a useable and accessible format?

Yes

Are the protocols appropriate and is the work technically sound?

Yes

Reviewer Expertise:

genomics, ornithology, evolution

I confirm that I have read this submission and believe that I have an appropriate level of expertise to confirm that it is of an acceptable scientific standard, however I have significant reservations, as outlined above.
